# Analysis of Structural Design Variations in MEMS Capacitive Microphones

**DOI:** 10.3390/s25030900

**Published:** 2025-02-02

**Authors:** Tzu-Huan Peng, Huei-Ju Hsu, Jin H. Huang

**Affiliations:** 1 Ph.D. Program of Mechanical and Aeronautical Engineering, Feng Chia University, Taichung 407102, Taiwan; p0800013@mail.fcu.edu.tw; 2 Qsensing Microelectronics Co., Ltd., Shenzhen 518556, China; rubyhsu@qsensing.com; 3 Department of Mechanical and Computer-Aided Engineering, Feng Chia University, Taichung 407102, Taiwan

**Keywords:** micro-electro-mechanical system (MEMS), capacitive microphone, equivalent circuit modeling, sensitivity, signal-to-noise ratio (SNR), acoustic overload point (AOP), compressed air test (CAT)

## Abstract

Different microstructures significantly affect the acoustic performance of MEMS capacitive microphones, particularly in key specifications of interest. This paper presents several microstructures, including rib-reinforced backplates, suspended diaphragms, and outer vent holes. Three MEMS microphone designs were implemented to analyze the impact of these microstructures. Equivalent circuit models corresponding to each design were constructed to simulate specifications such as sensitivity, signal-to-noise ratio (SNR), and low corner frequency (LCF), which were validated through experimental measurements. Finite Element Analysis (FEA) was also employed to calculate the acoustic damping of certain microstructures, the mechanical lumped parameters of the diaphragm, and the pre-deformation of the MEMS structure. A compressed air test (CAT) was conducted to evaluate the mechanical reliability of microphone samples. The results of simulations and measurements indicate that rib-reinforced backplates effectively improve microphone reliability, increasing the pass rate in CAT. Compared to fully clamped diaphragms, suspended diaphragms exhibit higher mechanical compliance, which enhances SNR performance but reduces AOP. Outer vent holes can achieve similar functionality to diaphragm vent holes, but their impact on improving AOP requires further design and testing.

## 1. Introduction

Among the wide applications of MEMS transducers, audio has emerged as a particularly important field. MEMS microphones and speakers have revolutionized the electroacoustic transducer market by offering unique advantages over traditional alternatives. These advantages include their compact size, cost-effectiveness, and seamless integration with other electronic devices. As a result, MEMS transducers have become the preferred choice for audio applications across wearable devices, consumer electronics, automotive systems, and healthcare equipment. With the rising popularity of 4C products (computers, communications, consumer electronics, and car electronics) incorporating audio input, along with the increasing demand for miniaturization and cost-effectiveness in audio devices, the demand for MEMS microphones and speakers is expected to continue rising in the foreseeable future [1].

The prominence of voice control, driven by the rapid development of artificial intelligence (AI) and the Internet of Things (IoT), has further fueled the demand for advanced microphone performance. Microphones must deliver high-fidelity sound quality for phone calls and video recording, low output distortion to enhance the accuracy of signal processing algorithms, and reliability suited to environments closely integrated with human daily life.

In terms of sound quality parameters, the demands for higher performance are primarily addressed by improving the signal-to-noise ratio (SNR) and the acoustic overload point (AOP). The SNR is a key performance indicator, reflecting the quality of the microphone’s output based on its noise level. A higher SNR ensures better signal quality, even at low sound levels. Conversely, the AOP defines the sound pressure level at which maximum acceptable signal distortion occurs. Enhancing the AOP expands the range of sound pressures the microphone can transduce accurately and with high fidelity. To increase SNR and AOP, many studies have focused on diaphragm structures, such as corrugated [2,3], slit-edged [4], spring-supported [5,6], free-plate [7], and semi-constrained [8] diaphragms, to achieve higher compliance and better linearity. Other approaches with higher manufacturing costs include using dual-backplate [9,10] and dual-diaphragm [11,12] designs, which theoretically double the output at the MEMS component. Another approach to improving SNR is to reduce the acoustic resistance of the MEMS structure, thereby minimizing thermal acoustic noise generated by the resistance. Among these, the squeeze-film acoustic resistance from the backplate is a primary source, leading to ongoing research on backplate structures that lower or eliminate this resistance [13,14,15]. Since SNR and AOP are, respectively, related to the noise and distortion of the signal, some research has focused on the design of microphone amplifiers. This includes various CMOS-integrated circuits, such as source-follower [16], differential amplifiers [17], and other circuits’ techniques [18,19,20] aimed at reducing the noise floor and distortion of the microphone signal while maintaining low power consumption.

MEMS microphones installed in personal devices must be capable of withstanding drops and variations in temperature and humidity during usage. These aspects have been extensively studied in prior research [21,22,23]. Another testing topic that is less frequently discussed in academia is the high sound-pressure impact on microphones, which some manufacturers refer to as the compressed air test (CAT). Shubham et al. [24], in the only academic paper published on this topic, highlighted that reducing the volume of the microphone’s rear cavity can decrease the maximum sound pressure experienced by the diaphragm during the impact. Additionally, adding ribs to the diaphragm can enhance the microphone’s reliability in CAT.

Ongoing research seeks to explore novel MEMS capacitive microphone structures within reasonable manufacturing cost constraints, focusing on designs that achieve higher SNR, enhanced AOP, and improved reliability within a compact package size of 2.75 mm × 1.85 mm × 0.9 mm, thereby enabling continued innovation in voice-controlled devices and high-fidelity audio applications.

The presentation of this work follows the sequence outlined below. Section 2 introduces three structural designs of capacitive MEMS microphones to be compared and discussed in this article: S1, a typical design used as a reference; S2, a ribbed backplate design; and S3, which incorporates both a ribbed backplate and a suspended diaphragm. Section 3 models these microphone designs in 3D using the commercial FEA software, COMSOL Multiphysics 6.3, based on their layouts. Important mechanical and electrical parameters, such as mass, compliance, and static capacitance, are determined using coupled Solid Mechanics and Electrostatics modules. Equivalent circuit models (ECMs) are then constructed for these microphone designs to simulate the microphone’s frequency response, thermal noise curve, and SNR. Section 4 briefly describes the manufacturing process of the MEMS microphones and Section 5 details the measurements conducted in an anechoic chamber to obtain the frequency response, SNR, AOP, and CAT. The measured results are then compared and analyzed against the simulated data, with further discussion on the impact of different structural designs on acoustic performance. Finally, the key findings of this study are summarized in Section 6.

## 2. The Designs of the Microphones

**Figure 1:**
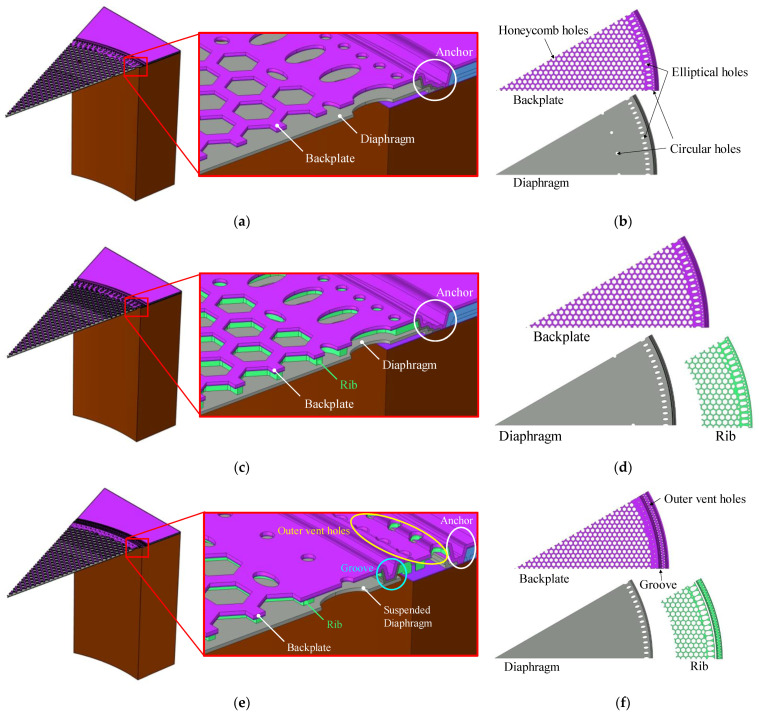
S1 design with a fully clamped diaphragm and no reinforced backplate. S2 design with a fully clamped diaphragm and a rib-reinforced backplate. S3 design with a suspended diaphragm and a rib-reinforced backplate: For the S1, S2 and S3 designs, respectively, (**a**,**c**,**e**) are sectional oblique views of the 3D FEA model at a 30-degree angle, along with localized magnifications near the anchor; (**b**,**d**,**f**) detail the layers.

This article presents three designs, S1, S2, and S3, to examine the impact of different structural configurations on the acoustic performance of MEMS capacitive microphones. Figure 1 provides sectional oblique views of designs S1, S2, and S3, respectively, along with detailed information about each layer. The main features of the different designs are described below.

S1 is a typical design with a fully clamped diaphragm boundary, featuring a radius of 367 μm. The perforated backplate has densely arranged honeycomb-shaped vent holes, each with an inscribed radius of 9 μm and a spacing of 12 μm. The air gap between the diaphragm and the backplate is 2.8 μm, with the boundaries of both fixed to the substrate by the anchor.

Based on the design of S1, S2 incorporates a 1 μm-thick rib-reinforced structure beneath the backplate near the anchor area. Stress concentrations are likely to occur near the edges of the backplate when the MEMS microphone is subjected to high sound pressure or airflow impacts [25]. Therefore, the rib structure in S2 is added to increase stiffness, enabling the backplate to better withstand high-pressure airflow impacts.

S3 features a rib-reinforced backplate along with a suspended diaphragm, which has a smaller radius of 360.5 μm. The edge of the diaphragm is attached to a groove around the periphery of the backplate, making it appear suspended. Compared to a fully clamped diaphragm, the suspended diaphragm allows its boundaries to rotate slightly in response to sound pressure, thereby enhancing its compliance.

All designs include a circular arrangement of elliptical vent holes near the diaphragm’s edge. These vent holes are used to adjust the pressure on the diaphragm and control the stress distribution at its edge. Furthermore, S3 includes an extra set of vent holes on the backplate, referred to as “outer vent holes,” located between the suspended diaphragm and the anchor. In terms of acoustic effects, they are expected to guide part of the airflow directly toward the rear cavity, thereby enhancing the microphone’s ability to withstand high sound pressure and improving its reliability and AOP. In fact, outer vent holes are also a necessary structure in the manufacturing of S3, and the related details will be explained in Section 4.

## 3. Equivalent Circuit Model

### 3.1. Sensitivity

**Figure 2:**
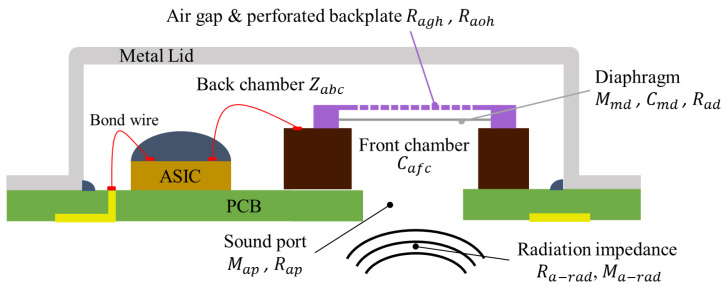
A cross-sectional diagram of the packaged capacitive MEMS microphone.

Figure 2 shows the cross-sectional diagram of the packaged capacitive MEMS microphone used in this study. The diagram illustrates the sequential path of sound propagation, which includes the impedances of acoustic radiation ($R_{a\text{-}rad},M_{a\text{-}rad}$), sound port ($M_{ap}$, $R_{ap}$), front chamber ($C_{afc}$), diaphragm ($M_{md}$, $C_{md}$), perforated backplate ($R_{agh}$, $R_{aoh}$), and back chamber ($Z_{abc}$). Actually, the backplate also vibrates due to the disturbance of sound waves, but this characteristic only becomes apparent in the ultrasonic frequency range [26]. In this study, only the audible frequency range is analyzed, and the backplate is considered non-vibrating. The corresponding lumped parameters are labeled in the figure as well.

**Figure 3:**
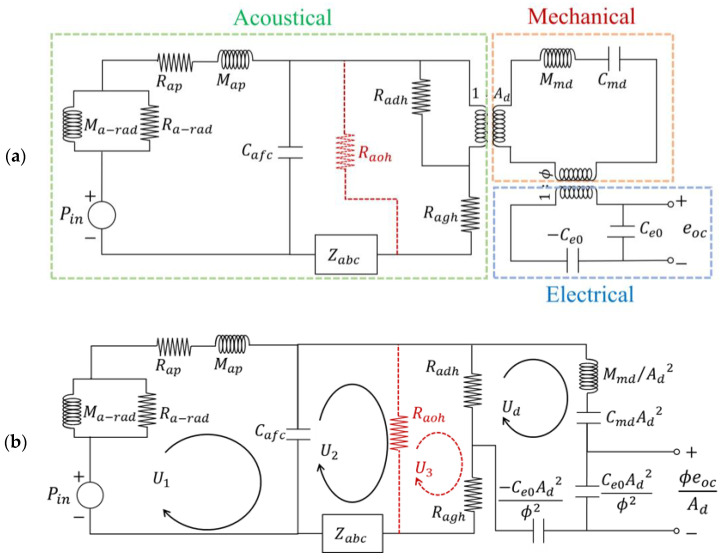
Equivalent circuit models of S1, S2 (without a red dashed line) and S3 (with a red dashed line): (**a**) separate domains (mechanical, electrical, and acoustical) coupled by transformers; (**b**) all domains are integrated into the acoustical domain.

**Figure 4:**
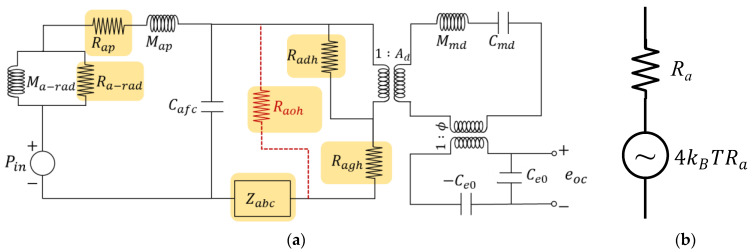
(**a**) Thermal noise-generating components marked with a highlighted box; (**b**) Thevenin equivalent circuit for thermal noise.

Figure 3a illustrates the proposed equivalent circuit models for the S1, S2, and S3 MEMS microphones, incorporating input pressure $P_{in}$, output voltage $e_{oc}$, effective area $A_{d}$, transduction factor ϕ, and static capacitance between the diaphragm and the backplate $C_{e0}$. These models take into account the sequential paths of sound propagation, as depicted in Figure 3. In Figure 4, The resistor on the red dashed line represents the acoustic resistance of the outer vent holes, which is included in the circuit model only for analyzing the design of S3, but not for S1 and S2.

To analyze microphone sensitivity, the equivalent circuit integrates components from different domains into the acoustical domain, as shown Figure 3b. Within each circuit loop, the flow is denoted by volume velocity $U_{i}$. Through circuit analysis, the sensitivity of the S1 and S2 microphones can be obtained as

(1) $$\frac{e_{oc}}{p}=\frac{\phi }{j\omega C_{e0}}\;\frac{1}{A_{d}}\;\left[\begin{array}{ccc} 0 & 0 & 1 \end{array}\right]{\left[\begin{array}{ccc} Z_{11} & -Z_{12} & 0\\ -Z_{12} & Z_{22} & -Z_{23}\\ 0 & -Z_{23} & Z_{33} \end{array}\right]}^{-1}\left[\begin{array}{c} 1\\ 0\\ 0 \end{array}\right]$$

where

(2) $$\begin{array}{l} Z_{11}=\left(R_{a\text{-}rad}//j\omega M_{a\text{-}rad}\right)+R_{ap}+j\omega M_{ap}+1/\left(j\omega C_{afc}\right),\;\\ Z_{12}=1/\left(j\omega C_{afc}\right),\;\;Z_{22}=1/\left(j\omega C_{afc}\right)+R_{adh}+R_{agh}+1/\left(j\omega C_{abc}\right),\\ Z_{23}=R_{adh},\;\;Z_{33}=R_{adh}+j\omega M_{md}/{A_{d}}^{2}+1/\left(j\omega C_{md}{A_{d}}^{2}\right) \end{array}$$

Similarly, the sensitivity of the S3 microphone can be obtained as

(3) $$\frac{e_{oc}}{p}=\frac{\phi }{j\omega C_{e0}}\;\frac{1}{A_{d}}\;\left[\begin{array}{cc} \begin{array}{cc} 0 & 0 \end{array} & \begin{array}{cc} 0 & 1 \end{array} \end{array}\right]{\left[\begin{array}{cccc} Z_{11} & -Z_{12} & 0 & 0\\ -Z_{12} & Z_{22} & -Z_{23} & 0\\ 0 & -Z_{23} & Z_{33} & -Z_{34}\\ 0 & 0 & -Z_{34} & Z_{44} \end{array}\right]}^{-1}\left[\begin{array}{c} \begin{array}{c} 1\\ 0 \end{array}\\ \begin{array}{c} 0\\ 0 \end{array} \end{array}\right]$$

where

(4) $$\begin{array}{l} Z_{11}=\left(R_{a\text{-}rad}//j\omega M_{a\text{-}rad}\right)+R_{ap}+j\omega M_{ap}+1/\left(j\omega C_{afc}\right),\\ Z_{12}=1/\left(j\omega C_{afc}\right),\;Z_{22}=1/\left(j\omega C_{afc}\right)+R_{aoh}+1/\left(j\omega C_{abc}\right),\\ Z_{23}=R_{aoh},\;Z_{33}=R_{aoh}+R_{adh}+R_{agh},\\ Z_{34}=R_{adh},\;Z_{44}=R_{adh}+j\omega M_{md}/{A_{d}}^{2}+1/\left(j\omega C_{md}{A_{d}}^{2}\right) \end{array}$$

### 3.2. Thermal Noise

Thermal noise is one of the main sources of self-noise in MEMS microphones [27]. The power spectral density (PSD) of this noise is related to the acoustical impedance as:

(5) $${\tilde{p}}_{n}^{2}=4k_{B}TRe\left[Z_{a}\right]\;\;\;\left({Pa}^{2}/Hz\right)$$

where $k_{B}$ represents the Boltzmann constant, T is the absolute temperature, and $Re\left[Z_{a}\right]$ denotes the real part of the acoustical impedance.

To calculate the thermal noise of the capacitive MEMS microphone, two modifications are made to the equivalent circuit depicted in Figure 3. First, the sound pressure source in Figure 3 is removed, as illustrated in Figure 4a. Second, each acoustic resistance in the circuit, highlighted with colored boxes, is replaced with the acoustic resistance connected in series with the noise-generating source. This modified circuit is referred to as the Thevenin equivalent circuit, as illustrated in Figure 4b.

Using the circuit in Figure 4a and employing a similar calculation method as for the microphone sensitivity, we can obtain the corresponding electrical noise $e_{n_{i}}$ (in $V/\sqrt{Hz}$) generated by the *i*th acoustic resistance. Since the noises from each source are independent and non-coherent signals, they are summed in an energy manner to obtain the total electrical noise:

(6) $$e_{n_{total}}^{2}=\sum_{i=1}^{N}e_{n_{i}}^{2}\;\;\;\;\;\left(V^{2}/Hz\right)$$

To obtain the overall noise level, the total electrical noise is A-weighted and then integrated over the entire frequency range (20 Hz to 20 kHz). This integration yields the noise level, expressed as:

(7) $$noise\;level=10\log_{10}\int_{20}^{20k}{\left[{e_{n}}_{total}\cdot w_{A}(f)\right]}^{2}df\;\left(dBA\right)$$

Subtracting the noise level from the sensitivity of the microphone gives the SNR:

(8) SNR=Sensitivity−noise level (dB)

### 3.3. Lumped Parameters

In the following sections, the methods for determining the parameters in the equivalent circuit model are briefly introduced. For components with simple geometries or regular patterns, the parameters can be calculated using analytical or numerical expressions derived from previous research, whereas complex structures require FEA. The material properties used in the FEA are listed in Table 1, and the electroacoustic parameters used in the equivalent circuit model and their values are presented in Table 2.

#### 3.3.1. Acoustic Radiation Impedance

External sound waves enter the MEMS microphone through the sound port on the PCB. The radiation impedance between the sound port and the external air is approximated to that of a vibrating disk on an infinite baffle [28]:

(9) $$R_{a\text{-}rad}=\frac{128}{9\pi^{2}}\frac{\rho_{0}c}{A_{p}},\;\;M_{a\text{-}rad}=\frac{8}{3\pi }\frac{\rho_{0}a_{p}}{A_{p}}$$

where $a_{p}$ is the radius of the sound port, $A_{p}(=\pi {a_{p}}^{2})$ is the port area, $\rho_{0}$ is the density of air, and c is the speed of sound in air.

#### 3.3.2. Acoustic Impedance of the Sound Port

In the case of laminar flow inside a small-radius port, the viscosity term of the Navier–Stokes equation dominates. The acoustic resistance and mass of fluid in the sound port are given by [29]:

(10) $$R_{ap}=\frac{8\mu L_{p}}{\pi {a_{p}}^{4}}\sqrt{1+\frac{\rho_{0}\omega {a_{p}}^{2}}{32\mu }},\;\;M_{ap}=\frac{\rho_{0}L_{p}}{\pi {a_{p}}^{2}}\left[1+{\left(3^{2}+\frac{\rho_{0}\omega {a_{p}}^{2}}{2\mu }\right)}^{-1/2}\right]$$

where $L_{p}$ is the length including end corrections, μ is the viscosity coefficient of fluid, and ω is angular frequency.

#### 3.3.3. Air-Gap and Backplate-Vent-Hole Resistance (Damping)

Kaczynski et al. presented an analytical model for the viscous damping in the air gap and perforation holes [30]. The model is divided into three distinct regions. The first region, $R_{G}$, represents the flow resistance caused by the squeezed-film effect in the air gap. This aligns with Skvor’s previously proposed model [31]. The second region, $R_{H}$, refers to the vertical flow resistance within the perforation hole. The third region, $R_{I}$, accounts for the flow resistance in the intermediate area that connects the air gap and the perforation hole. The overall flow resistance is determined by summing up these three contributions:

(11) $$R_{agh}=R_{G}+R_{H}+R_{I}$$

where

(12) $$R_{G}=\frac{12\mu }{\pi h^{3}N}\left(\frac{\varepsilon }{2}-\frac{\varepsilon^{2}}{8}-\frac{\ln\varepsilon }{4}-\frac{3}{8}\right),\;\;R_{H}=\frac{\mu }{r_{1}^{3}N}\;\left(a_{1}\left(\varepsilon \right)\frac{L}{r_{1}}+a_{2}\left(\varepsilon \right)\right),\;\;R_{I}=\frac{\mu }{r_{1}^{3}N}\;\left(\frac{b_{1}\left(\varepsilon \right)}{\left(h/r_{1}\right)}+\frac{b_{2}\left(\varepsilon \right)}{{\left(h/r_{1}\right)}^{2}}+\frac{b_{3}\left(\varepsilon \right)}{{\left(h/r_{1}\right)}^{3}}\right)$$

Here, μ is the viscosity of air, h is the air-gap distance, N is the number of holes in the perforated backplate, $\varepsilon ={2\pi \sqrt{3}\left(r_{1}/d_{0}\right)}^{2}/3$ is the porosity of the perforated plate, $r_{1}$ is the hole radius, and $d_{0}$ is the distance between the centers of the two holes. The coefficients $a_{i}\left(\varepsilon \right)$ and $b_{i}\left(\varepsilon \right)$ are given by

(13) $$\begin{array}{l} a_{1}\left(\varepsilon \right)=2.53,\;\;a_{2}\left(\varepsilon \right)=2.9736-0.5009\varepsilon -0.2403\varepsilon^{2}-1.0415\varepsilon^{3}\\ b_{1}\left(\varepsilon \right)=-1.2279+3.9286\varepsilon -8.3047\varepsilon^{2}+10.364\varepsilon^{3}-4.7769\varepsilon^{4}\\ b_{2}\left(\varepsilon \right)=1.8564-4.5354\varepsilon +6.26363\varepsilon^{2}-6.1491\varepsilon^{3}+2.6235\varepsilon^{4}\\ b_{3}\left(\varepsilon \right)=-0.091428+0.39734\varepsilon -0.89348\varepsilon^{2}+0.97977\varepsilon^{3}-0.39857\varepsilon^{4} \end{array}$$

This model is applicable under certain conditions, including the Knudsen number <0.1, the porosity ε<0.9, the ratio between air gap of the two plates and the perforation hole radius $h/r_{1}>0.1$, and the squeeze number $\sigma =\omega (12\mu {r_{2}}^{2})/(h^{2}p_{0})\ll 1$, where $p_{0}$ represents ambient pressure and $r_{2}=r_{1}/\sqrt{\varepsilon }$.

#### 3.3.4. Acoustic Impedance of the Back Chamber

The compression and expansion of the air within small chambers are typically assumed to occur rapidly compared to the rate of thermal diffusion. This allows the process to be treated as adiabatic, meaning no heat transfer occurs between the air and chamber walls. Under this assumption, the acoustic impedance is $Z_{a}=1/\left(j\omega C_{a}\right)$, where $C_{a}$ is the adiabatic compliance of the air volume.

However, when heat transfer does happen at the chamber walls, it results in a thermal boundary layer and significant temperature variations within the chamber. For a MEMS microphone, the enclosure walls are often metals with much higher thermal conductivity than air. Consequently, the chamber walls act as isothermal boundaries, effectively maintaining a relatively constant temperature. When the thermal boundary layer becomes sufficiently large relative to the chamber dimensions, which occurs for small chambers and at low frequencies, the compression and expansion of air within the chamber transitions from adiabatic to isothermal. This means that the air no longer behaves as an ideal gas, and the acoustic impedance of the chamber must be corrected.

Kuntzman and LoPresti introduced a thermal correction term, $Z_{t}$, which is added in parallel with the adiabatic acoustic impedance, $Z_{a}$, to obtain the modified acoustic impedance of the chamber as [32]:

(14) $$Z_{abc}=\frac{1}{Z_{a}^{-1}+Z_{t}^{-1}}$$

where

(15) $$Z_{a}=\frac{1}{j\omega C_{a}}=\frac{\gamma p_{0}}{j\omega V},\;Z_{t}=\frac{1}{j\omega C_{a}\left(\gamma -1\right)\left(1-\frac{\bar{T}}{T_{a}}\right)}$$

with

(16) $$\frac{\bar{T}}{T_{a}}=1-\frac{tanh\beta a}{\beta a},\;\beta =\sqrt{\frac{j\omega \rho_{0}c_{p}}{\kappa }}$$

where γ is the ratio of specific heats for gas, $c_{p}$ is the specific heat at constant pressure of the gas inside the chamber, $p_{0}$ is the environmental pressure, $\rho_{0}$ is the static density, V is the volume of the chamber, $\bar{T}$ is the spatial average of the amplitude of temperature oscillation in the chamber due to the acoustic excitation, $T_{a}$ is the oscillation amplitude of the temperature under adiabatic conditions, a is half the height of the chamber, and κ is the thermal conductivity of the gas in the cavity. Substituting (15) and (16) into (14), the modified acoustic compliance of the back chamber is obtained as follows:

(17) $$Z_{abc}=\frac{1}{j\omega C_{a}\left[1+\left(\gamma -1\right)\left(\frac{tanh\beta a}{\beta a}\right)\right]}$$

#### 3.3.5. The Transduction Factor

The transduction factor represents the conversion ratio between voltage and mechanical force, and in the circuit, it ensures the compatibility of units between the electrical and acoustic domains. The expression for the transduction factor, ϕ, is given by [33]:

(18) $$\phi =-\frac{Q_{0}}{h}=-\frac{C_{e0}e_{b}}{h}$$

where $Q_{0}$ is the charge accumulated on the electrodes (diaphragm and backplate), h is air-gap distance, $C_{e0}$ is the static electrical capacitance.

#### 3.3.6. Damping of the Diaphragm and Outer Vent Holes

**Figure 5:**
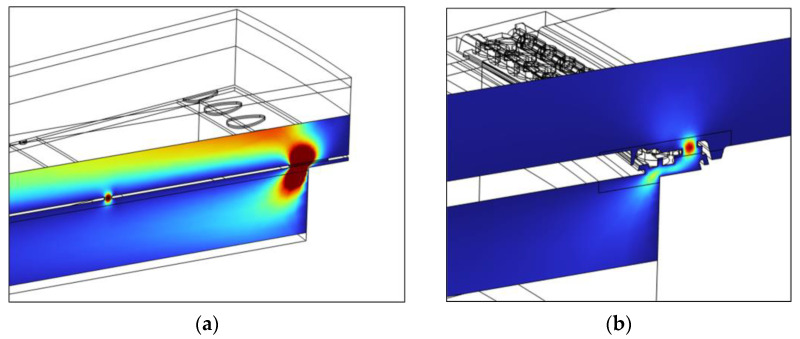
The cross-sectional velocity fields from the FEM models at 1 kHz: (**a**) The diaphragm vent holes for S1; (**b**) the outer vent holes for S3. The red regions indicate higher acoustic velocity, while the blue regions indicate lower acoustic velocity.

Since the diaphragm vent holes and the outer vent holes are not regularly arranged over a large area and have varying shapes, developing a closed-form expression for their acoustic resistance is challenging. Therefore, the Thermoviscous Acoustic module in COMSOL Multiphysics 6.3 is used to determine the damping. To ensure efficient calculations, a 10-degree section model with a limited thickness range is constructed, including the vent holes of interest. All solid structures are assumed to be rigid, and only the air domain is modeled. The lower and upper boundaries of the model are defined as the output and input ports for the plane sound wave, respectively. The simulated cross-sectional velocity fields of the diaphragm vent holes and the outer vent holes are shown in Figure 5. The acoustic resistance is calculated as follows:

(19) $$R_{adh}\;or\;R_{aoh}=\frac{P_{diss}}{\frac{1}{2}{\left|U\right|}^{2}}$$

here, $P_{diss}$ denotes the total dissipated power, obtained by integrating the total power dissipation density over the volume, and U is the volume velocity through the vent holes.

$R_{adh}$ plays a key role in controlling the lower corner frequency $F_{LC}$, the point at which the frequency response drops to −3 dB relative to the sensitivity. This is an important specification, as it limits the microphone’s low operating frequency due to the RC high-pass filter formed by $R_{adh}$ and $C_{abc}$ in the equivalent circuit. In a typical MEMS microphone design, such as those of S1 and S2, $F_{LC}$ can be approximated as follows [34]:

(20) $$F_{LC}\approx \frac{1}{2\pi R_{adh}C_{abc}}$$

$R_{aoh}$, which is only in the S3 design, functions similarly to $R_{adh}$. In combination with $R_{adh}$ and $C_{abc}$, $R_{aoh}$ also forms a high-pass filter, though with a more complex relationship to $F_{LC}$.

#### 3.3.7. Mechanical Properties of Diaphragm

The effective mass and compliance of a fully clamped circular diaphragm have analytical expressions [4]. However, for diaphragms with complex shapes or boundary conditions, such as the suspended diaphragm in this study, the Solid Mechanics module in COMSOL Multiphysics 6.3 is used for calculations. When a uniform sound pressure p is applied to the diaphragm, it undergoes deformation. The effective area is defined as

(21) $$A_{d}=\frac{\sum w_{i}A_{i}}{w_{max}}$$

where $w_{i}$ and $A_{i}$ represent the displacement and area of the ith element, respectively, and $w_{max}$ denotes the maximum displacement of the diaphragm. The effective acoustic properties of the diaphragm can be calculated as

(22) $$C_{ad}=\frac{\sum w_{i}A_{i}}{p},\;M_{ad}=\frac{1}{{\left(2\pi f_{0}\right)}^{2}C_{ad}}$$

where $f_{0}$ represents the diaphragm’s first resonance frequency, determined through eigenfrequency analysis using FEA software, COMSOL Multiphysics 6.3. Then, using the impedance relationship between the acoustic and mechanical domains, the mechanical parameters of the diaphragm are determined as follows

(23) $$C_{md}=\frac{C_{ad}}{{A_{d}}^{2}},\;M_{md}={A_{d}}^{2}M_{ad}$$

#### 3.3.8. Static Electrical Capacitance Under Bias Voltage

**Figure 6:**
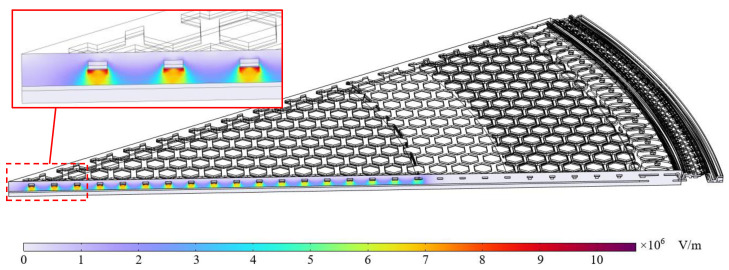
The cross-sectional electric field from the FEM models for the S3 microphone design.

The static capacitance formed by the perforated backplate and diaphragm under bias is also obtained using COMSOL Multiphysics 6.3 by coupling the Solid Mechanics and Electrostatics modules. For computational efficiency, a 30-degree section model of the diaphragm and backplate is constructed, surrounded by a 9 μm-thick air domain. The bias voltage $e_{b}$ for different microphone designs is applied to the backplate electrode, while the diaphragm is grounded at 0 V. The simulated cross-sectional electric field for S3 is shown in Figure 6. The static capacitance is calculated as follows:

(24) $$C_{e0}=\frac{Q_{0}}{e_{b}}$$

where $Q_{0}$ denotes the accumulated charge on the electrode, calculated from surface integral of the surface charge density.

## 4. Fabrication

All MEMS microphone designs were fabricated using the same process to ensure consistent manufacturing costs. Special structures such as the rib-reinforced backplate and suspended diaphragm were created through specific photomask patterns; however, the detailed photomask designs will not be disclosed here.

**Figure 7:**
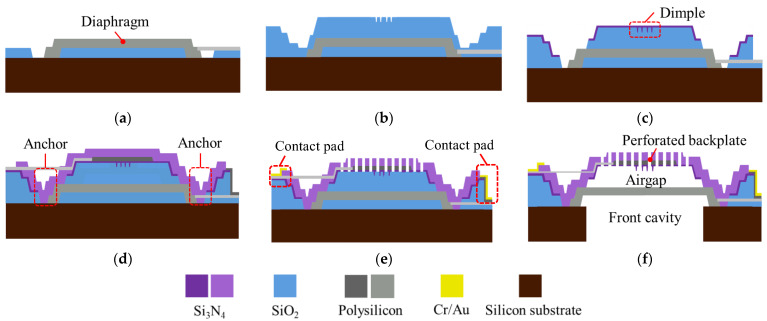
A simplified fabrication flow for fabricating the capacitive MEMS microphone with the S1 design, in steps (**a**–**f**).

A simplified fabrication process, represented by the S1 design, is shown in Figure 7. The substrate material is an 8-inch n-type single-crystal silicon wafer with a single-side polished (SSP) surface, a thickness of 725 μm, and a bulk resistivity of 2.5 Ω-cm. Firstly, the based sacrificial layer deposition is followed by polysilicon diaphragm deposition and etch (Figure 7a). The next sacrificial layer deposition will form the air gap between the diaphragm and backplate, and the shape of anti-stick dimples are defined (Figure 7b). A thin layer of Si3N4 is then deposited to form the dimples, followed by etching the location of the anchor (Figure 7c). After that, polysilicon is deposited and patterned to form the electrodes of the backplate, and a thick layer of Si3N4 is deposited (Figure 7d). The backplate is patterned to form vent holes, and Cr/Au is deposited to form metal contact pads (Figure 7e). Turn the entire MEMS component upside down, and use deep-reactive ion etch (DRIE) to form the front cavity. Finally, all sacrificial oxides are removed by a buffered oxide etch (BOE) process (Figure 7f).

**Figure 8:**
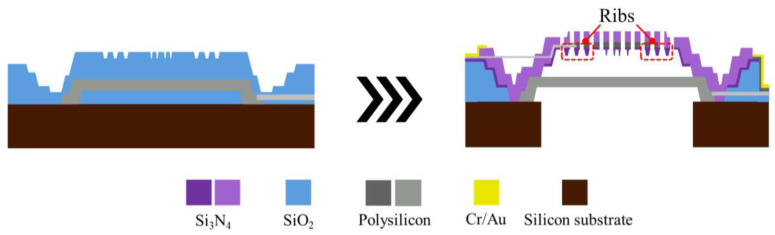
Fabrication flow features for the capacitive MEMS microphone with the S2 design.

**Figure 9:**
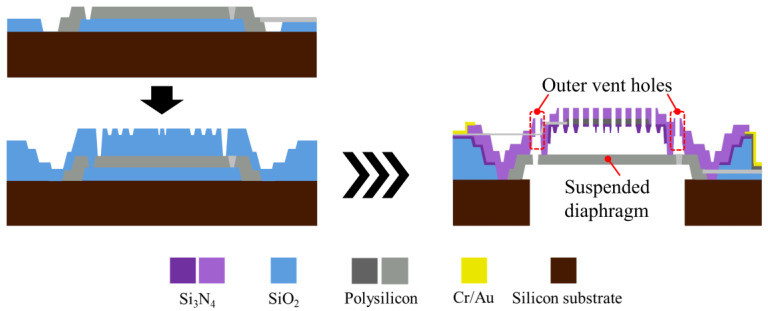
Fabrication flow features for the capacitive MEMS microphone with the S3 design.

Figure 8 and Figure 9 illustrate the fabrication flow features for S2 and S3, respectively. For the S2 design, the method for creating the rib is similar to that for the dimple: the rib’s shape is etched onto the oxide sacrificial layer, and the rib is formed during the deposition of the backplate. For S3, the key difference lies in the diaphragm edges, which are not fixed to the anchor positions as in S1 and S2. After the diaphragm is initially deposited, an annular etching pattern is applied to create the unanchored circular plate, leaving a small unetched area for electrode connection. Subsequently, an oxide sacrificial layer is deposited on top of the diaphragm, and the areas where the backplate will connect to the diaphragm are etched. The suspended diaphragm structure is then formed during the subsequent backplate deposition process.

Additionally, it should be noted that the outer vent holes in the S3 design are crucial. If the backplate lacks outer vent holes, during the final step of the process, BOE can only etch the oxide layer between the suspended diaphragm and the anchor from the bottom. This would result in the incomplete removal of the oxide layer in that area during the usual etching time. If the BOE is left in for longer, other structures may be damaged. Therefore, outer vent holes are added between the suspended diaphragm and the anchor on the backplate, allowing BOE to enter from the backplate side to etch that area and ensure complete removal of the oxide layer.

The completed MEMS components will eventually be integrated with an analog application specific integrated circuit (ASIC) and assembled into a 2.75 mm × 1.85 mm × 0.9 mm package for a bottom-port microphone.

**Figure 10:**
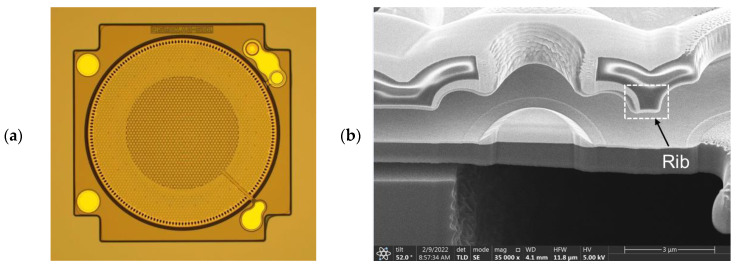

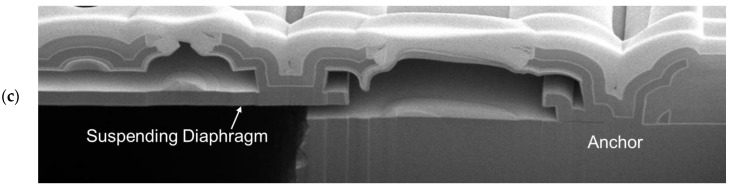
Images of critical features of the proposed MEMS microphone design: (**a**) The MEMS die for S1 by OM; (**b**) the rib-reinforced backplate for S2 by TEM; (**c**) the suspended diaphragm for S3 by TEM.

An optical microscope image (OM) of the MEMS die for S1 is shown in Figure 10a. Figure 10b,c are the images of the detailed MEMS structures of the rib-reinforced backplate for S2 and the suspended diaphragm for S3, respectively, captured using a transmission electron microscope (TEM).

## 5. Measurement and Simulation

### 5.1. Frequency Response, SNR, and AOP

**Figure 11:**
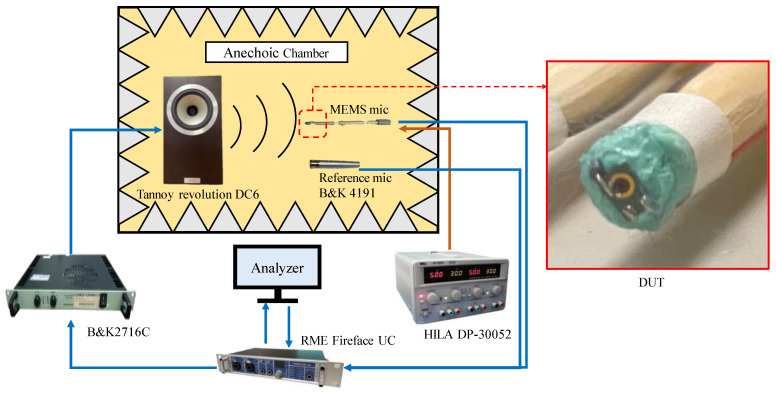
Experimental setup for frequency response, SNR, and AOP measurement.

The frequency response, SNR, and AOP were measured for six MEMS microphones with the S1, S2, and S3 designs in an anechoic chamber. Then, the measurements for each design were averaged to obtain the measured data. The measurement process followed the flow chart depicted in Figure 11.

For frequency response measurements, a test signal of a swept sine wave ranging from 50 Hz to 10 kHz is used. The microphone device under test (DUT) is mounted on one end of a wooden stick using clay. Its sound inlet hole is aligned horizontally with the center of the speaker diaphragm and positioned 50 cm away. The test signal is played through the speaker, producing a 1 Pa sound wave at the DUT’s location, which is received by the microphone and converted into an electrical signal.

For SNR measurements, no signal is played by the speaker. Instead, the A-weighted noise level of the DUT is measured. Then, the SNR is calculated through Equation (8), where sensitivity is the value at 1 kHz obtained in frequency response measurements.

For AOP measurements, the speaker emits a 1 kHz tone with gradually increasing sound pressure. When the total harmonic distortion (THD) of the DUT reaches 10%, the sound pressure level measured by the reference microphone besides the DUT is recorded as the AOP for that DUT.

**Figure 12:**
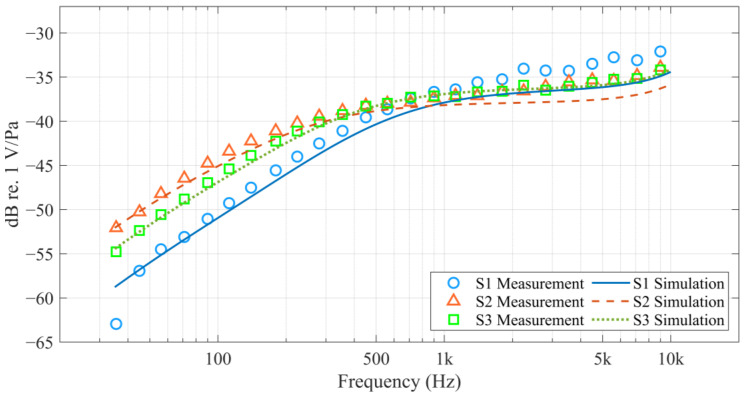
Frequency response experimental data (markers) and simulation results (curves) for S1, S2, and S3.

The frequency response experimental data and simulation results for all designs are presented in Figure 12. The simulation results generally match the experimental data, particularly in the low-frequency range (below 1 kHz), where they align very closely. However, in the high-frequency range (above 1 kHz), there is more discrepancy, which may be due to the model ignoring the acoustic mass of various MEMS structures and the packaged PCB’s baffle effect.

Table 3 lists the experimental data and simulation results for four important specifications of MEMS microphones, including sensitivity, lower corner frequency, SNR, and AOP (no simulation results for AOP). One thing to note is that, to facilitate the comparison of the frequency response for each design, we adjusted the bias voltage so that the sensitivity of the different designs is approximately −37 dBV/Pa. The bias values used in each design are listed in Table 2 and follow the order S1 > S2 > S3. This is due to two factors. The first factor is the pre-deformation of the MEMS components, which is caused by residual stress in the materials. Table 4 shows the pre-deformation at the center of the diaphragm and backplate for each design without bias voltage, as well as the calculated air-gap distances. It points out that designs with reinforced backplates, namely S2 and S3, exhibit larger downward deformation of the backplate, resulting in smaller air-gap distances. According to the theoretical model derived in Section 3.1, the sensitivity of a MEMS microphone is proportional to the transduction factor ϕ. Moreover, ϕ is proportional to the bias voltage and inversely proportional to the air-gap distance, as shown in Equation (18). Consequently, for the S1 design to achieve the same sensitivity as S2 and S3, a higher bias voltage is required. The second factor is the compliance of the diaphragm. From the mechanical compliance by COMSOL Multiphysics 6.3 shown in Table 2, S3’s suspended diaphragm has the highest compliance among the three designs, which means that under the same acoustic driving force, S3’s diaphragm exhibits the largest displacement—giving S3 the highest ’mechanical sensitivity’. Since S2 and S3 have similar air-gap distances, S2 requires a higher bias voltage to achieve the same sensitivity as S3.

In terms of the lower corner frequency $F_{LC}$, S1 has the highest value, followed by S3 and S2. This trend can be attributed to the vent-hole structures in each design. S1’s diaphragm has the most vent holes, indicating the lowest acoustic resistance, which, according to Equation (20), results in the highest $F_{LC}$. Although S2 has more vent holes on its diaphragm than S3, the numerous outer vent holes along the edge of S3’s backplate ultimately cause S3’s $F_{LC}$ to be slightly higher than that of S2.

**Figure 13:**
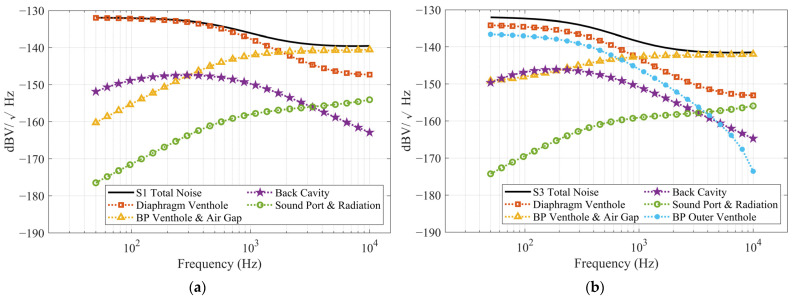
The simulated noise spectrum density including each MEMS component and package noise contributions: (**a**) for S1; (**b**) for S3.

The simulated SNR of the MEMS microphone, obtained using the method described in Section 3.2, excludes ASIC noise. As a result, the simulated values are higher than the measured ones; however, since the same ASIC is used, their trends remain consistent. The factors influencing SNR are quite complex, but in this study, one primary factor can be identified: the bias voltage. Observing the noise spectrum density of S1 and S3 in Figure 13, apart from the additional noise contribution from the outer vent holes in S3, the trends of the remaining noise components are very similar to those of S1. However, S3 exhibits overall lower noise levels than S1. This is because these noise components are amplified as the bias voltage increases. Consequently, when the bias voltage of S1 is raised to achieve a sensitivity comparable to that of S3, its noise levels are significantly amplified as well.

AOP is a key specification used to evaluate how loud a microphone can operate. Excluding the impact of the ASIC, it primarily refers to the amplitude of the diaphragm’s linear vibration. Typically, a sudden increase in nonlinear components occurs when the diaphragm makes contact with the backplate. As a result, designs with a larger air gap or lower diaphragm compliance tend to exhibit higher AOP. Accordingly, the measured AOP results, which follow the order S1 > S2 > S3, can also be reasonably explained by the two factors related to the bias voltage part discussed earlier. Since S1 has a larger air gap compared to S2 and S3, the diaphragm has a greater linear vibration range under high pressure. Furthermore, the diaphragm of S2 is stiffer than that of S3, resulting in smaller diaphragm displacement and fewer nonlinear components under the same sound pressure drive.

From the above analysis, in our microphone designs (S1, S2 and S3), SNR and AOP exhibit opposite trends. This indicates that with changes in the factors of air-gap and diaphragm compliance, there is a trade-off relationship between SNR and AOP.

The package size of 2.75 mm × 1.85 mm × 0.9 mm is considered exceptionally small for commercial microphone products nowadays. Smaller package sizes pose greater challenges in terms of both sound quality and manufacturing. Table 5 compares the performance of this work with commercial products of the same package size and type. The larger discrepancy in LCF for this work compared to commercial products is attributed to the greater number of vent holes designed in the diaphragm. However, the SNR and AOP of this work are not significantly behind those of commercial products.

### 5.2. Compressed Air Test

**Figure 14:**
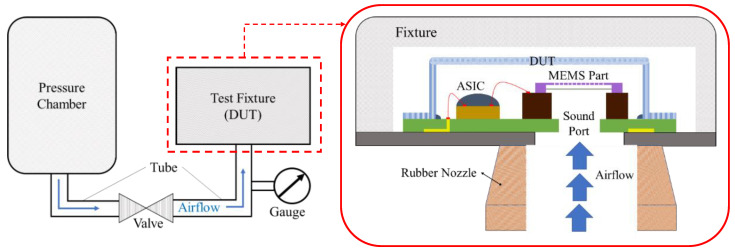
The diagram of the experimental test setup for CAT.

The compressed air test is a method designed to simulate scenarios where the microphone’s sound inlet port is rapidly covered by objects or tapped by fingers, exposing the microphone to a high-pressure air pulse. With the growing prevalence of wearable and close-fitting electronic devices, this test has become increasingly significant for MEMS microphones and is a key specification for evaluating microphone reliability. To conduct the test, the MEMS microphone sample is positioned on a test fixture with an air tube aligned to its sound port. The air supply is controlled by a solenoid valve, which delivers a brief, high-pressure airflow of approximately 0.8 MPa to the microphone. Figure 14 illustrates a simplified diagram of the test setup and the test fixture with the sample in place. After the test, the microphone’s frequency response will be evaluated. If any abnormalities are detected, such as deviations beyond the specified range, the MEMS components will be further examined using an optical microscope to determine the cause of the failure.

**Figure 15:**
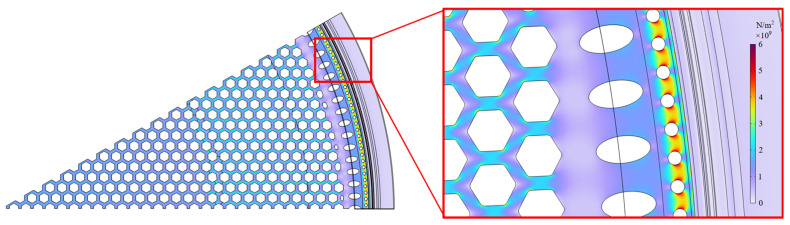
The static stress distribution for the S1 design, modeled by applying an upward pressure of 0.8 MPa to the bottom of the backplate.

Since CAT is a dynamic airflow event in which the diaphragm and backplate of the microphone deform nonlinearly within an extremely short time, accurately modeling the entire CAT process is challenging and computationally intensive. As a compromise, a static loading simulation of the backplate is performed using FEA software to observe the stress distribution and determine the maximum stress for different designs. Using the Solid Mechanics module in COMSOL Multiphysics 6.3, an upward static pressure of 0.8 MPa is applied to the bottom of the backplate. Figure 15 illustrates the simulated stress distribution on the backplate of S1 design under static loading, showing that stress concentrations occur around the circular vent holes near the edge. However, it must be noted that compared to dynamic analysis, static analysis lacks considerations of velocity and acceleration, thereby overlooking the effects of inertial and damping forces. As a result, the accuracy of the analysis results should be approached with caution.

The simulated maximum von Mises stress on the backplate under a static pressure of 0.8 MPa and the CAT experimental results for S1, S2, and S3 are presented in Table 6. In the stress analysis, it was observed that the maximum stress of S1, which lacks a rib-reinforced backplate, reached 6.26 GPa. This value exceeds the fracture strength of the stiffest material in the backplate, Si3N4, which is 5.87± 0.62 GPa [39]. In contrast, the maximum stresses in S2 and S3, both featuring rib-reinforced backplates, remained below the fracture strength of Si3N4. Furthermore, CAT experiments showed that S1 had a pass rate of only 38%, while both S2 and S3 achieved a 100% pass rate. These results not only align with the simulation findings but also confirm that the rib-reinforced design significantly improves the reliability of the backplate.

## 6. Conclusions

This article investigates structural design variations in MEMS capacitive microphones, comparing their impact on acoustic performance and reliability. Three microphone designs—S1, S2, and S3—were proposed and implemented. S1 features a typical fully clamped diaphragm with no reinforced backplate, S2 incorporates a fully clamped diaphragm with a rib-reinforced backplate, and S3 adopts a suspended diaphragm with a rib-reinforced backplate.

Theoretical models were established to predict the acoustic characteristics of MEMS microphones, which were then validated through experimental measurements. The analysis of simulation results and measurement data confirms that a rib-reinforced backplate significantly improves microphone reliability. However, it also increases backplate pre-deformation, reducing the air-gap distance. While this reduction allows for a lower bias voltage, it comes at the expense of a decreased AOP. Similarly, the suspended diaphragm, a MEMS structure with higher diaphragm compliance, enables lower bias voltages but further reduces the AOP. In-depth analysis reveals that among microphones with the same sensitivity, those with lower bias voltage can achieve higher SNR.

Additionally, the study explores the effects of vent-hole quantity on the diaphragm. In theory, vent holes in the diaphragm reduce the acoustic pressure load, thereby enhancing the microphone’s reliability and AOP, albeit at the cost of a low-frequency roll-off in the frequency response. In the S3 design, the presence of outer vent holes is essential for the fabrication process. These holes have a similar effect to diaphragm vent holes but allow airflow to pass directly into the back cavity without involving the diaphragm. However, the impact of outer vent holes on improving AOP remains unclear and requires further comparison with additional designs.

In this work, the trade-off between SNR and AOP is evident with changes in the factors of air gap and diaphragm compliance. Compared to existing commercial microphones of the same size, the performance of the proposed microphone in this study is not far behind, demonstrating its potential for further development. Optimizing various parameters to achieve better SNR and AOP remains a key objective for ongoing development and future research.

## Figures and Tables

**Table 1:** The material properties used in FEA software.

Material	Young’s Modulus (GPa)	Poisson’s Ratio	Density $\left(kg/m^{3}\right)$	Internal Stress (MPa)
Monocrystalline silicon	170	0.28	2329	0
Polycrystalline Silicon	160	0.22	2320	30
Si_3_N_4_	250	0.23	3100	280
SiO_2_	70	0.17	2200	−200

**Table 2:** Electroacoustic parameters of designs S1, S2, and S3.

Parameters	Description	Unit	S1	S2	S3
$M_{a\text{-}rad}$	Acoustic radiation mass	$10^{2}\;kg/m^{4}$	2.59	2.59	2.59
$R_{a\text{-}rad}$	Acoustic radiation resistance	$10^{10}\;N\cdot s/m^{5}$	1.21	1.21	1.21
$M_{ap}$	Acoustic mass of sound port (1 kHz)	$10^{3}\;kg/m^{4}$	8.81	8.81	8.81
$R_{ap}$	Acoustic resistance of sound port (1 kHz)	$10^{7}\;N\cdot s/m^{5}$	5.95	5.95	5.95
$R_{adh}$	Acoustic resistance of vent holes on DP * (1 kHz)	$10^{10}\;N\cdot s/m^{5}$	1.20	3.50	3.60
$R_{agh}$	Acoustic resistance of air gap and vent holes on BP ** (1 kHz)	$10^{9}\;N\cdot s/m^{5}$	2.30	2.51	2.52
$R_{aoh}$	Acoustic resistance of outer vent holes of BP ** (1 kHz)	$10^{10}\;N\cdot s/m^{5}$	--	--	5.80
$C_{afc}$	Acoustic compliance of front chamber	$10^{-15}{\;m}^{5}/N$	1.09	1.06	1.13
$C_{abc}$	Acoustic compliance of back chamber	$10^{-14}{\;m}^{5}/N$	1.80	1.80	1.80
$M_{md}$	Effective mass of DP *	$10^{-10}\;kg$	2.23	2.35	2.18
$C_{md}$	Effective compliance of DP *	$10^{-2}\;m/N$	1.23	1.01	1.49
$A_{d}$	Effective area	$10^{-7}{\;m}^{2}$	1.85	1.91	1.79
$C_{e0}$	Static capacitance	$10^{-13}F$	4.86	5.84	5.62
$e_{b}$	Bias voltage	Volt	16	12.5	11
ϕ	Transduction factor	$10^{-6}\;C/m$	−3.91	−4.23	−3.53

* DP stands for ‘Diaphragm’. ** BP stands for ‘Backplate’.

**Table 3:** Important acoustic specifications of all microphone designs.

	**S1**	**S2**	**S3**
	Experiment		
Sensitivity @ 1 kHz (dBV/Pa)	−36.45	−37.23	−37.06
$\mathrm{Lower}\;\mathrm{corner}\;\mathrm{frequency}\;F_{LC}$ (Hz)	450	224	280
SNR ^1^ (dBA)	53.09	60.77	60.95
AOP (dBSPL)	127.94	124.96	124.43
	Simulation		
Sensitivity @ 1 kHz (dBV/Pa)	−37.88	−38.17	−36.92
$\mathrm{Lower}\;\mathrm{corner}\;\mathrm{frequency}\;F_{LC}$ (Hz)	447	215	322
SNR ^1,2^ (dBA)	58.94	62.26	64.00

^1^ Frequency range evaluated: 50 Hz~10 kHz. ^2^ The contribution from the ASIC noise is not accounted for in the SNR simulation.

**Table 4:** Experimental and simulated pre-deformation and air-gap distance at the center position of the MEMS components under unbiased conditions for all microphone designs.

	**S1**	**S2**	**S3**
	White Light Interferometry (WLI)		
Diaphragm (μm)	0.11	−0.1	−0.96
Backplate (μm)	−0.256	−0.81	−1.475
Air Gap * (μm)	2.434	2.09	2.285
	FEA, COMSOL Multiphysics 6.3		
Diaphragm (μm)	−0.034	−0.029	−0.550
Backplate (μm)	−0.528	−0.931	−1.348
Air Gap * (μm)	2.306	1.898	2.002

* The original air gap is 2.8 μm in manufacturing settings.

**Table 5:** Comparison with commercial products with the same package size of 2.75 mm × 1.85 mm × 0.9 mm.

Manufacturer (Model)	Microphone Type	LCF (Hz)	Sensitivity dBV/Pa	SNR (dBA)	AOP (dBSPL)
Goertek [35] (S15OB381-124)	Bottom port	12	−38	62.5	128
TDK InvenSense [36] (MMICT4076-00-908)	Bottom port	85	−38	63	127
Knowles [37] (SPV61A0LR5H-1)	Bottom port	35	−40	66	133
Knowles [38] (SPV0142LR5H-1)	Bottom port	85	−38	62.5	124
This work **(**S3**)**	Bottom port	280	−37.06	60.95	124.43

**Table 6:** Simulated maximum stress and CAT experimental results of design S1, S2, and S3.

	S1	S2	S3
Simulation: Max. von Mises stress (GPa) ^1^	6.26	3.68	3.34
Experiment: Pass rate of CAT ^2^	38%	100%	100%

^1^ Applying upward pressure of 8 MPa on the backplate. ^2^ More than 500 pieces of each design are tested.

## Data Availability

Dataset available on request from the authors.
